# Superior temporal gyrus functional connectivity predicts transcranial direct current stimulation response in Schizophrenia: A machine learning study

**DOI:** 10.3389/fpsyt.2022.923938

**Published:** 2022-08-05

**Authors:** Animesh Kumar Paul, Anushree Bose, Sunil Vasu Kalmady, Venkataram Shivakumar, Vanteemar S. Sreeraj, Rujuta Parlikar, Janardhanan C. Narayanaswamy, Serdar M. Dursun, Andrew J. Greenshaw, Russell Greiner, Ganesan Venkatasubramanian

**Affiliations:** ^1^Alberta Machine Intelligence Institute, University of Alberta, Edmonton, AB, Canada; ^2^Department of Computing Science, University of Alberta, Edmonton, AB, Canada; ^3^Schizophrenia Clinic, Department of Psychiatry, National Institute of Mental Health and Neuro Sciences, Bengaluru, India; ^4^Translational Psychiatry Laboratory, Neurobiology Research Centre, National Institute of Mental Health and Neuro Sciences, Bengaluru, India; ^5^Canadian VIGOUR Centre, University of Alberta, Edmonton, AB, Canada; ^6^Department of Psychiatry, University of Alberta, Edmonton, AB, Canada

**Keywords:** transcranial direct current stimulation (tDCS), Schizophrenia, auditory verbal hallucinations, resting-state functional connectivity, machine learning, treatment response

## Abstract

Transcranial direct current stimulation (tDCS) is a promising adjuvant treatment for persistent auditory verbal hallucinations (AVH) in Schizophrenia (SZ). Nonetheless, there is considerable inter-patient variability in the treatment response of AVH to tDCS in SZ. Machine-learned models have the potential to predict clinical response to tDCS in SZ. This study aims to examine the feasibility of identifying SZ patients with persistent AVH (SZ-AVH) who will respond to tDCS based on resting-state functional connectivity (rs-FC). Thirty-four SZ-AVH patients underwent resting-state functional MRI at baseline followed by add-on, twice-daily, 20-min sessions with tDCS (conventional/high-definition) for 5 days. A machine learning model was developed to identify tDCS treatment responders based on the rs-FC pattern, using the left superior temporal gyrus (LSTG) as the seed region. Functional connectivity between LSTG and brain regions involved in auditory and sensorimotor processing emerged as the important predictors of the tDCS treatment response. L1-regularized logistic regression model had an overall accuracy of 72.5% in classifying responders vs. non-responders. This model outperformed the state-of-the-art convolutional neural networks (CNN) model—both without (59.41%) and with pre-training (68.82%). It also outperformed the L1-logistic regression model trained with baseline demographic features and clinical scores of SZ patients. This study reports the first evidence that rs-fMRI-derived brain connectivity pattern can predict the clinical response of persistent AVH to add-on tDCS in SZ patients with 72.5% accuracy.

## Introduction

Schizophrenia (SZ) is one of the top 10 disabling disorders, afflicting 1% of the world’s population (1). Antipsychotic medications constitute the mainstream treatment for SZ. Nonetheless, about 30% of the patients have treatment-resistant symptoms, despite two or more antipsychotic treatments (other than clozapine) (2). In such patients with treatment-resistant SZ, clozapine is recommended as the drug-of-choice (3); however, only about 30–60% of these treatment-resistant patients respond to clozapine (4, 5). Several alternative avenues like brain stimulation are being evaluated to treat this challenging clinical condition in SZ. Several brain stimulation techniques like Electroconvulsive Therapy (ECT) (6, 7), Transcranial Magnetic Stimulation (TMS) (8, 9) and transcranial Direct Current Stimulation (tDCS) (10, 11) offer promising adjuvant options to treat persistent symptoms in SZ. Meta-analyses of brain stimulation studies for treating persistent symptoms in SZ have supported their clinical utility (7, 8, 10, 11).

Nonetheless, striking inter-individual variation in the clinical response is common across all these brain stimulation techniques. Hence, there has been recent interest in applying data-driven, machine learning (ML) approaches to produce models that can accurately predict each patient’s response to brain stimulation treatments to enable better treatment decisions (12–17). ML approaches are data-driven strategies that make fewer assumptions than classical statistical methods and focus on prediction instead of hypothesis testing; they identify relevant patterns for the prediction task and often allow the incorporation of expert knowledge (18).

Earlier ML studies in SZ produced models that could accurately predict response to ECT using pre-treatment resting-state functional Magnetic Resonance Imaging (fMRI) (16), resting-state electroencephalography (EEG) (15), or multi-modal MRI data (14). Likewise, in Major Depressive Disorder (MDD), other ML analyses produced tools that could predict clinical response to neuromodulation treatment using baseline parameters like (i) EEG power spectra (12), (ii) baseline blood-oxygen-level-dependent (BOLD) activity (13), or (iii) phenotypic (clinical), demographic, and neuropsychological data (17). Structural MRI-derived models have also been shown to predict treatment response following tDCS-paired cognitive training in healthy older adults (19). These ML studies used several different classification methods, including support vector machine (SVM), extreme learning machine, linear discriminant analysis, least absolute shrinkage and selection operator (LASSO), gradient boosting algorithm, and random forest classifier. These supervised methods identify patterns that can effectively distinguish categories (like responders vs. non-responders) and determine which treatment may be best for an individual patient (18).

While several ML techniques may predict which patients will benefit from ECT and TMS, very few consider identifying patients who respond to tDCS. Most of these studies have used ML to predict which MDD patients will benefit from tDCS; we are unaware of any ML studies describing learned models that predict clinical response to tDCS in SZ patients. Note that tDCS is increasingly reported as effective for treating positive (20, 21) and negative (10, 22) SZ symptoms. As with other neuromodulation treatment scenarios, considerable inter-individual variability in clinical responses to tDCS in SZ is a major challenge. This has motivated our research to understand which moderator variables may potentially influence response to tDCS in SZ; this may help to predict treatment effects and to determine neurostimulation treatment parameters that can achieve stable and durable (and thus clinically relevant) results (22).

Clinically translatable differentiation of tDCS responders from non-responders will help clinicians identify the appropriate treatment for individual patients. Toward this effect, we propose utilizing ML approaches to produce a model for predicting tDCS outcomes for SZ patients with persistent auditory verbal hallucinations (AVH) based on baseline resting-state functional connectivity (rs-FC) brain imaging data. Motivating this ML approach, as noted earlier, is the wide variability in patient biology which means different patients require different neuromodulation protocols to produce clinically meaningful therapeutic effects (23) thereby necessitating a precision medicine approach (24). In this context, rs-FC measures, which reflect statistically relevant BOLD temporal connections among spatially distinct regions within the human brain, present information that might help predict a specific patient’s response to neuromodulation therapies. ML algorithms can produce models that can find patterns in intrinsic brain activity to develop rs-FC-based models that distinguish treatment responders from non-responders in psychiatric disorders (25, 26). As machine-learned analyses can combine many features to predict an outcome of clinical importance, they are suitable for the translational goal of producing models to accurately predict whether an individual patient will benefit from tDCS (25, 26).

In SZ, tDCS-induced reduction in AVH has been shown to be associated with pathophysiologically relevant changes in the rs-FC within the AVH-brain network (27). Hence, in this study, we examined the feasibility of identifying SZ patients with persistent AVH (SZ-AVH) who will respond to tDCS treatment based on resting-state functional MRI (rs-fMRI) acquired before treatment. Our study analyzes the baseline rs-FC data of 39 SZ-AVH who received add-on treatment with conventional tDCS (*N* = 31) or High-Definition tDCS (*N* = 8). Conventional tDCS delivers very-low-intensity direct current (typically ∼2 mA) to underlying cortical regions by the placement of relatively large bio-conductive electrodes on the scalp (28). High Definition tDCS (HD-tDCS) uses much smaller electrodes (12 mm) arranged in a concentric 4 × 1 ring configuration to achieve a more precise stimulation effect in terms of focality (29) and polarity effects (30). The clinical data of tDCS/HD-tDCS effects in these patients (37 of 39 patients) were published earlier (31–33); however, the brain imaging data of these patients have not been reported. In this study, using state-of-the-art ML algorithms, we developed a model using the pre-treatment rs-FC data that can predict the clinical outcome (i.e., response vs. non-response) to add-on tDCS treatment.

## Materials and methods

### Patient description

**FIGURE 1:**
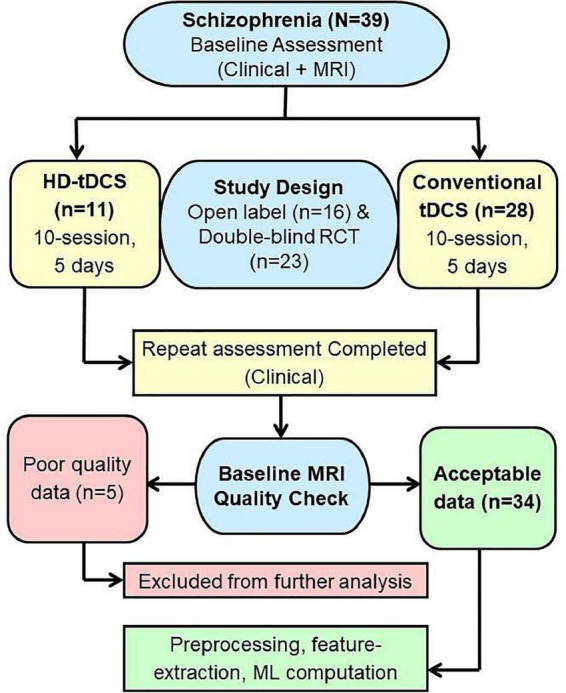
Study flow chart.

This study included 39 SZ patients with AVH (SZ-AVH) fulfilling DSM-IV criteria (34), right-handed, within the age range of 18–48 years, accessing clinical services at the National Institute of Mental Health And Neurosciences (NIMHANS), Bengaluru, India. Details regarding illness onset, course, and treatment response were collected from the patient and at least one first-degree relative (primary caregiver). The patients were recruited if they had refractory AVH, i.e., the persistence of AVH without remission despite treatment with at least one antipsychotic medication at an adequate dose for a minimum period of 3 months. The patients were maintained on the same medications throughout the study period. Also, patients were screened for the following exclusion criteria: psychiatric emergency, substance dependence, neurological disease, uncontrolled medical condition, pregnancy/post-partum status, and contraindication for tDCS (e.g., local lesion, metal in the head). All patients had given informed written consent as approved by the Institute Ethics Committee. The Research Ethics Board at the University of Alberta approved the secondary analysis of archived, de-identified data. We included patients that had completed the tDCS treatment course for whom the pre-tDCS MRI data was available. As we had to discard five subjects due to the poor scan quality, the final study sample comprised of 34 SZ patients (Figure 1).

### Clinical assessments

Mini International Neuropsychiatric Interview Plus (M.I.N.I.-Plus) was administered to ascertain the diagnosis of SZ (35). SZ symptoms were assessed by Scale for Assessment of Positive Symptoms (SAPS) (36) and Scale for Assessment of Negative Symptoms (SANS) (37). AVH was assessed by the Auditory Hallucination subscale of the Psychosis Rating Scale (PSYRATS-AH) (38). The primary outcome measure was change in the severity of AVH. Treatment responders were defined by at least a 25% reduction in the total PSYRATS-AH score after tDCS (conventional/high-definition); otherwise, patients were classified as non-responders.

### Transcranial direct current stimulation procedures

Thirty-nine SZ patients received either 10-sessions of conventional tDCS (32) or HD-tDCS (31) for 5 days as per previous descriptions further detailed in Supplementary material. Both involved cathodal stimulation of the left temporoparietal junction (L-TPJ) with a 2 mA current for 20 min. All of these patients tolerated the stimulation well and none reported any notable side-effects.

### Image acquisition

Imaging studies were done on the first day before initiating the tDCS. Neuroimaging data were acquired from one of the two 3.0 Tesla MRI scanners. Acquisition parameters are given below (For scanner-wise distribution of study sample, refer to Supplementary Table 1).

• *MRI Scanner-1:* Brain imaging data of 27 SZ patients (conventional tDCS) were acquired using the Magnetom Skyra 3T system (Siemens Healthineers, Erlangen, Germany) with the following parameters: *Structural MRI*: T1-weighted three-dimensional MRI was performed (TR = 8.1 ms, TE = 3.7 msec, nutation angle = 8°, FOV = 256 mm, slice thickness = 1-mm without inter-slice gap, NEX = 1, matrix = 256 × 256) yielding 165 sagittal slices. *Resting-State fMRI*: BOLD sensitive echo-planar imaging was obtained using a 32-channel coil yielding dynamic scans (153 scans for seven subjects and 303 scans for twenty subjects). The scan parameters were: TR = 2,000 ms; TE = 30 msec; flip angle = 78° degree; slice thickness = 3-mm; Slice order: Descending; Slice number = 37; Gap = 0.75 mm; Matrix = 64 × 64, FOV = 192 × 192, voxel = 3.0-mm, isotropic.
• *MRI Scanner-2*: Brain imaging data for 12 SZ patients (11 HD-tDCS; 1 conventional tDCS) were acquired using the Ingenia CX 3T system (Philips Healthcare, Best, Netherlands) with the following parameters: Structural MRI: T1-weighted three-dimensional MRI (TR = 6.5 msec, TE = 2.9 msec, nutation angle = 9°, FOV = 256 mm, slice thickness = 1-mm without inter-slice gap, NEX = 1, matrix = 256 × 256) yielding 192 slices. *Resting-state fMRI*:—BOLD-sensitive echo-planar imaging (TR = 2,200 ms, TE = 28 ms, flip angle = 80°, slice thickness = 3-mm, slice order = ascending, slice number = 44, gap = 0.3 mm, matrix = 64 × 62, FOV = 211 × 211, voxel = 3.3-mm, isotropic) was obtained with a 32-channel coil yielding 275 dynamic scans.

### Image processing

Both structural and functional neuroimaging data were processed using the CONN toolbox (version 18b).^1^ The image processing steps involved AC-PC correction, realignment, slice-time correction, detection of outlier scans (ART-based; thresholded at the 99th percentile), brain tissue segmentation into gray matter/white matter/CSF, normalization to MNI space, and smoothing (4-mm Gaussian kernel). After pre-processing, visual quality control (QC) for MNI boundary registration was performed for both structural and functional images. Wherever the registration was poor—e.g., if brain areas fell outside the MNI boundary or significant overlap between gross anatomy was absent—registration was re-attempted, and the instance was discarded if the boundary mismatch persisted.

In accordance with the recommended CONN-data processing pipeline (39), first-level covariates for each subject’s rs-fMRI data included head motion time-series (composite motion threshold ≥ 2 mm) and ART-based “scrubbed” artifact of global signal fluctuations (scan-to-scan global signal z-value threshold ≥9). This was followed by aCompCor-based denoising where linear regression of these confounding effects was performed to obtain CSF and white matter masked blood oxygen level-dependent (BOLD) time-series. Visual QC was done for the effect of denoising. The resulting BOLD signal was band-pass filtered (0.008–0.09 Hz), de-spiked and linear detrended. For the clinical and pediatric population, up to 30% scrubbing has been found to be permissible (40), so subjects with ≥30% dynamics censored were excluded from further analyses. Out of 39 subjects’ data, two subjects’ data had to be discarded due to incorrigible boundary registration issues and three subjects’ data were dropped because their rs-fMRI had ≥30% invalid scans. The final study sample comprised of 34 SZ patients.

### Machine learning

Features were extracted using seed-based functional connectivity with the left superior temporal gyrus (LSTG) as the seed region (i.e., for computing the correlations between this region and other brain regions) using a 15 mm radius sphere around the MNI coordinate (–48, 0, 0) (41). The choice of LSTG seed region is driven by two factors: (a) this region’s role in auditory hallucination pathophysiology as supported by neuroimaging evidence (41), and (b) it being a close approximation of the site of cathodal direct current stimulation (see Supplementary material). Additionally, this chosen seed region is inclusive of brain areas like Insula—a brain area implicated in salience—a feature crucial to the pathophysiology of AVH, and thus likely to capture aberrant connectivity patterns contributing to the experience of AVH.

We computed the Pearson correlation between the mean time-series of seed region with individual time-series from all other voxels in the brain. The correlation values were then normalized using Fisher Z-transformation. This procedure yielded a scalar feature value for each voxel of the brain, generating a 3D feature matrix of size 91 × 109 × 91 for the single LSTG seed-point. We extracted feature values from voxels belonging to *apriori* selected regions of the brain [as determined by Harvard cortical and subcortical atlases]^2^ based on their neurobiological basis in the pathogenesis of auditory hallucinations (42–45). These regions are listed in Supplementary Table 1 with supporting references. We used an L1 regularized logistic regression algorithm to train our classifier with the default hyperparameters that are provided by Scikit-Learn^3^, then evaluated the performance of the model using five shuffled iterations of 10-fold balanced (for class) cross-validation based on accuracy, specificity, sensitivity, and precision. 10-fold cross-validation is a standard machine learning technique where we randomly divide our observations into 10 groups, or folds, of approximately equal size and class distribution. The first fold is treated as a test set, and the model is trained on the remaining 9 folds while making sure that no data-leakage has occurred. As a state-of-art comparative method, we also tried the convolutional neural network (CNN) algorithm on whole-brain LSTG-connectivity first with, then without, transfer learning (46–48). In order to directly compare the performance of our models using paired *t*-test, we used the same metrics and cross-validation folds for training and testing, as the proposed L1 regularized logistic model.

CNNs have become a state-of-the-art method for solving various prediction tasks in computer vision (48–50). However, CNNs are often ineffective when trained on smaller datasets (51, 52). The generalization ability of the CNNs strongly depends on the size of the training data and the CNN architecture’s complexity. If we train a CNN model using smaller training data with randomized weights initialization, the model might have high variance and very high error on a test set. This situation is prevalent in clinical problems; for example, in our case, we have data from only 34 tDCS-treated patients for training and evaluating the model. This is why we used the transfer learning method, anticipating it would help to handle this problem. Transfer learning is often used to learn a model for a “target” domain when we have a limited number of training instances for that domain, but have many training instances for a related “source domain.” We have borrowed the transfer learning idea from deep learning-based computer vision applications (53), which is now widely applied in various domains. Researchers have pre-trained models with natural photographs or medical images for applications such as respiratory disease classification (54), early-stage skin cancer (55), early glaucoma diagnosis (56), brain image (MRI and CT) segmentation (57), cancer classification (58), acute intracranial hemorrhage (59), musculoskeletal abnormality detection (60), and cell segmentation (61). This motivated us to use the transfer learning method of first pre-training our CNN models on a disjoint dataset, and then subsequently continued to train them on our target dataset of 34 tDCS subjects. This often produces a better representation of fMRI features in lower layers of CNN, which then would yield better initialization of model parameters for training on the target task (62).

For pre-training, we used a disjoint dataset of resting-state fMRI images from several cohorts, including healthy controls, SZ, obsessive-compulsive disorder (OCD), and unaffected first-degree relatives of SZ patients (FDR-SZ). The clinical global impression scores (CGI) (63) of subjects during the time of fMRI acquisition were used as labels for the pre-training task: we assigned the 186 healthy controls and 62 FDR-SZ with CGI score = 0 the label of 0, and assigned the 44 SZ and 149 OCD patients who were at least moderately ill (CGI score >3) the label of 1. This yielded a total of 441 instances: 248 and 193 for classes 0 and 1, respectively. We oversampled instances in the minority class based on sex and age to balance the classes, producing 496 samples for pre-training. We chose CGI as the label for this pre-training task to capture global changes in fMRI signals that are associated with psychiatric illnesses in general. The choice of CGI index is motivated by its ability to indicate clinically meaningful subject status across psychiatric diagnoses and is related to our target task of predicting the treatment response. CGI score has been previously used to predict therapeutic outcome of cognitive intervention in sample of youth with heterogeneous psychiatric diagnoses (64). However, note that CGI was used only for the pre-training task in a disjoint dataset, as a way to “initialize” the parameters—and so this does not directly influence the analyses of treatment effects in the study cohort. (In general, the sample complexity only involves the specific target data). Imaging and clinical assessments for these cohorts, as well as the study subjects, were conducted in the same medical center (NIMHANS). Note that this pre-training process did not use any subjects from the target dataset.

**FIGURE 2:**
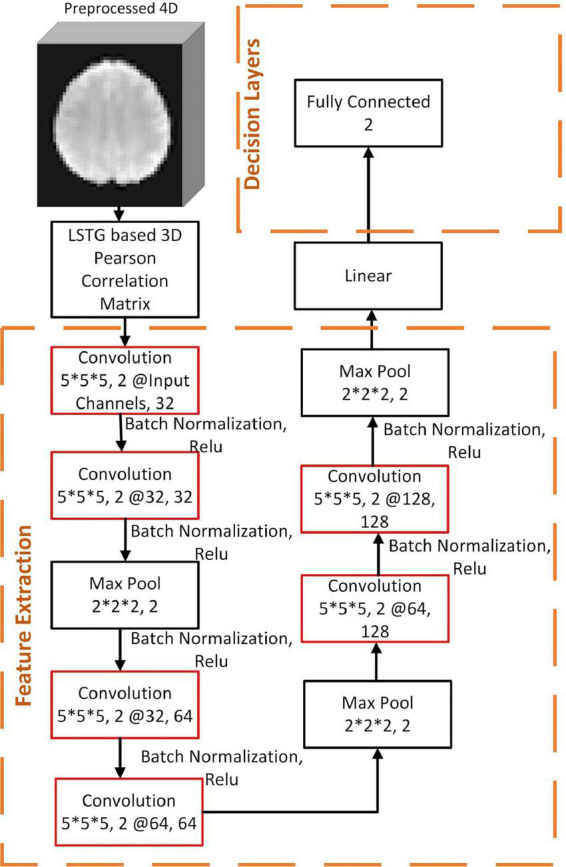
Convolutional Neural Network Models for prognosis prediction.

Figure 2 shows our CNN architecture where each convolution layer has 4 parameters depicted in order—kernel size, padding, input channel numbers and the number of filters. This is followed by a max-pooling layer with 2 parameters—kernel size and stride. After the feature extraction layers, we linearized the extracted features and then passed to a fully connected layer with 1 parameter—the number of output units. The model takes the LSTG seed-point-based 3D Pearson correlation matrix as a fixed input size of 91 × 109 × 91 for each subject.

After pre-training with 496 instances, we froze the parameters in the first four layers of the feature extraction, replaced the remaining decision layers, and then continued to train on the target dataset and target label. We report results for our CNN models—both with and without pre-training (Table 2). For each CNN model, we used cross-entropy as a loss function and used Adam optimizer with a learning rate 0.0001. To reduce the risk of overfitting, 50% of layers were dropped out during the training time. Also, we used a maximum of 1,000 epochs to train our models, with early-stopping criteria for 100 epochs—i.e., we calculated the validation error after each training epoch, and if the error was found to be not decreasing for a span of 100 epochs, then the training state was reverted back by 100 epochs. Models were implemented in PyTorch (v1.0.1) (65), and trained on a computer with Intel(R) Xeon(R) Platinum 8168 CPU, 32GB RAM and a 32GB Tesla V100-SXM2 GPU.

**TABLE 1:** Demographic table for responders (*n* = 17) and non-responders (*n* = 17).

Characteristic	Responder (*M* ± *SD*)	Non-responders (*M* ± *SD*)	Statistic	*P*
Age	30.06 ± 7.89	32.23 ± 7.47	*t* = –0.80	0.429
Sex (Male: Female)	7:10	12:5	χ^2^ = 1.90	0.167
Years of education**^#^**	14.11 ± 1.71	13.70 ± 1.67	*t* = 0.68	0.496
Duration of untreated illness (months)**^#^**	12.88 ± 23.86	9.35 ± 15.14	*t* = 0.49	0.620
Total duration of illness (months)^#^	105.88 ± 88.39	106.23 ± 71.16	*t* = –0.01	0.990
Olanzapine equivalent^#^	15.68 ± 8.98	22.70 ± 16.67	*t* = –1.53	0.136
Pre SAPS^#^	44.47 ± 19.66	31.06 ± 12.36	*t* = 2.38	0.023
Pre SANS^#^	38.35 ± 20.89	16.94 ± 12.58	*t* = 3.61	0.001*
Pre MADRS^#^	13.47 ± 7.01	10.25 ± 5.67	*t* = 1.44	0.159
Pre PSYRATS-AH	31.0 ± 4.74	29.82 ± 5.84	*t* = 0.62	0.536
Post PSYRATS-AH	16.17 ± 6.09	26.76 ± 6.10	*t* = –4.90	<0.001*
%Improvement**^$^**	47.7 ± 17.0	10.4 ± 8.0		

**^#^**Values of these variables were missing for the same one subject; we imputed those values using mean value imputation.

*Significance thresholded at 0.05 (two-tailed).

**^$^**[Pre RCT Score–Post RCT score/Pre RCT Score] or [Post RCT score–Post Open-label Score/Post RCT Score].

**TABLE 2:** Performance of models using 5 × 10-fold Cross-validation—Mean (standard error).

	Accuracy	Precision	Sensitivity	Specificity	True positive	True negative	False positive	False negative
**L1 regularized—Logistic regression**	**72.5 (3.8)**	**74.5 (4.1)**	**78.0 (4.9)**	**67.0 (5.4)**	**13.0 (0.6)**	**11.2 (0.3)**	**5.8 (0.3)**	**4.0 (0.6)**
**CNN**	59.41 (1.93)	59.43 (1.90)	58.82 (4.07)	60.0 (3.07)	10.0 (0.69)	10.2 (0.52)	6.8 (0.52)	7.0 (0.69)
**Pre-trained CNN**	68.82 (1.05)	69.63 (1.90)	68.24 (4.27)	69.41 (3.86)	11.6 (0.72)	11.8 (0.65)	5.2 (0.65)	5.4 (0.72)

Bold indicates the best performing model.

## Results

The study sample had an even split of 17 responders (change in PSYRATS-AH score ≥ 25%) and 17 non-responders (change in PSYRATS-AH score < 25%), thereby yielding a chance-level prediction performance of 50%. Table 1 shows that the two groups did not differ on age, gender ratio, duration of untreated illness, total duration of illness, or baseline severity of AVH. The responder group had significantly higher baseline psychopathology (SAPS and SANS) scores compared to the non-responder group.

The proposed L1-regularized logistic regression model, over the LSTG connectivity features with voxels in brain regions implicated in AVH pathophysiology, yielded superior performance (accuracy = 72.5%) when compared to the classic CNN model (accuracy = 59.41%; paired *t*-test, *p* = 0.003) but was not significantly greater compared to the pre-trained CNN model (accuracy = 68.82%; paired *t*-test, *p* = 0.470) (Table 2). However, the CNN models, which incorporated LSTG connectivity with whole-brain voxels showed a more stable (lower variance in cross-validation) performance (Table 2). Further, the L1 logistic regression algorithm with neuroimaging features outperformed the L1 logistic regression algorithm developed using only the baseline demographic and clinical features (age, sex, years of education, duration of untreated illness, total duration of illness, Olanzapine equivalent, and baseline psychopathology scores) that provided an accuracy of 66.2% ± 3.9% (refer to Supplementary Table 2 for additional details).

**FIGURE 3:**
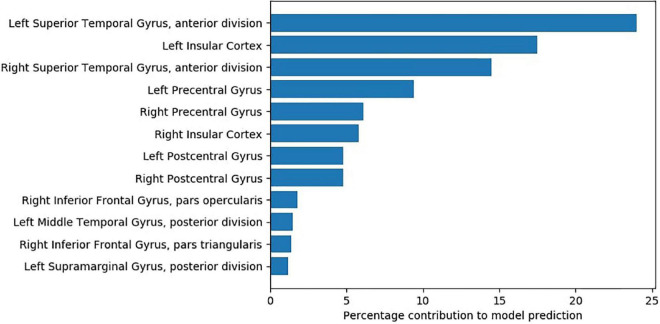
Percentage contributions of brain regions based on voxels with top 1000 SHAP values.

We used Shapley additive explanation values (SHAP) (66) to estimate the relative importance of features contributed by individual brain regions. Figure 3 lists the important regions. To identify these regions, we initially selected the 1,000 voxels that had the top SHAP values. We then computed the percentage contribution of each region—how many voxels from the selected 1,000 voxels belong to each region.

## Discussion

The present study used ML to produce a tool to predict improvement in persistent AVH with add-on tDCS therapy in SZ patients. Based on resting-state fMRI data, we used baseline (Pre-tDCS) rs-FC associated with LSTG to predict treatment response to add-on tDCS using a L1-regularized logistic regression, as well as more complex CNN. Two versions of CNN models were developed—one without any pre-training and another pre-trained on an independent dataset. In general, we observed that simpler logistic regression using a set of *a priori* target ROIs yielded superior results with an accuracy of 72.5%, precision of 74.5%, sensitivity of 78.0%, and specificity of 67.0%. However, we observed CNN models to be more stable with lower variance in prediction performance. This suggests the usefulness of simple pathophysiology-driven models (in this context, focus on LSTG as it is the site of auditory processing) for prognostic predictions in psychiatry.

The study findings show that a model that uses the rs-FC of LSTG with the following set of regions—postcentral gyrus, right inferior frontal gyrus, left middle temporal gyrus, left supramarginal gyrus—can accurately predict treatment response to add-on tDCS therapy for AVH. The most significant contribution in tDCS response prediction is from rs-FC within left STG and between left and right STG. This finding concurs with existing neuromodulation research in SZ-AVH. Higher cerebral blood flow to left STG is reported to distinguish SZ-AVH TMS responders from TMS non-responders (67). Tonically increased cerebral blood flow to left STG has been observed to persist in SZ-AVH patients even after treatment with TMS and reduction in AVH severity (68). Contextually, note an auditory processing study—where participants were required to detect voice embedded within short bursts of white noise—revealed that increasing excitability of left STG (with anodal tDCS) causes a significant increase in “false alarms” in healthy controls (69). As we administered cathodal tDCS/HD-tDCS to the area corresponding to left STG, it is possible that the application of cathodal tDCS led to a suppression of pre-tDCS hyperactivity of left STG; and this pre-tDCS activity level—indicated by rs-FC of left STG seed with left STG—contributed to the prediction of tDCS treatment response. Lastly, though left STG activation is more prominent during the occurrence of AVH—possibly suggesting that AVH could reflect internal speech originating in the left temporal lobe (70)—activation of its right homolog during AVH is also common (71).

Pre-tDCS rs-FC of LSTG seed with bilateral insular cortex, especially the left insular cortex, made the second highest contribution to predicting tDCS treatment response. The role of the insula in AVH pathophysiology has been well established in the literature (42, 72). AVH symptom-capture studies have reported increased cerebral activation in bilateral insula during the occurrence of AVH in SZ patients (73–75). Intrinsic connectivity (Degree Centrality) within the right insula, which is reflective of self-related processing deficits, appears to be significantly reduced in SZ-AVH compared to tinnitus patients and healthy controls (76). SZ-AVH patients were shown to have higher fractional Amplitude of Low-Frequency Fluctuations (fALFF) in insula than SZ patients without AVH (77). Interestingly, left fronto-temporoparietal tDCS was shown to reduce rs-FC of left TPJ with left anterior insula; notably, the magnitude of reduction of this TPJ-insula connectivity was correlated with the reduction in AVH severity after tDCS in SZ patients (27).

Other regions contributing to the tDCS response prediction model include bilateral precentral and postcentral gyri; both these regions show activation (73) and higher activation likelihood estimate during the occurrence of AVH (43, 44). The precentral gyrus is the site of the primary motor cortex responsible for controlling voluntary movements, and the supplementary motor cortex is responsible for planning voluntary motor actions (78). Contextually, it is noteworthy that both overt and covert speech (i.e., thinking) are amongst the most complex of motor acts (79). A weak efference copy of the intended overt and covert speech leads to corollary discharge dysfunction with resultant AVH in SZ (80). Moreover, after add-on treatment with fronto-temporoparietal tDCS, the strength of efference copy is shown to improve in SZ-AVH (81); this may mediate the therapeutic effects of tDCS (82). Thus, the relevance of the precentral gyrus in predicting the clinical response of AVH to tDCS may be understood in the context of the link between the precentral gyrus and efference copy.

The postcentral gyrus, the site of the primary somatosensory cortex (83), is responsible for integrating somatosensory stimuli and memory formation since it also houses the secondary somatosensory cortex (83). Note that the secondary somatosensory cortex subserves source-monitoring (84), and deficient source-monitoring underlies the pathogenesis of AVH (85). Add-on fronto-temporo-parietal tDCS ameliorates source-monitoring deficit in SZ patients with persistent AVH (86). Besides, the somatosensory cortex receives somatic sensations from the body, including sensory consequences of self-initiated actions (83). Thus, alongside the precentral gyrus, the somatosensory cortex is crucial to the execution of corollary discharge phenomena, thereby integral to the neurocircuitry of AVH (in addition to other brain areas involved in speech generation, perception, and integration).

This study observed a lesser contribution from pre-tDCS rs-FC of LSTG with right inferior frontal gyrus (IFG) to tDCS response prediction. Right IFG has been implicated in AVH pathophysiology in SZ patients (41, 75); moreover, rs-FC of right IFG with left TPJ decreased following treatment with left fronto-temporoparietal tDCS in SZ (although without correlating with a reduction in AVH severity) (27). Perhaps the role of the right IFG is integral to AVH pathophysiology in SZ and possibly contributory to tDCS treatment response; this warrants a closer examination by future studies. The left middle temporal gyrus and left supramarginal gyrus made a smaller contribution to the tDCS response prediction model. These regions have also been implicated in AVH symptom-capture in fMRI studies (73) as well as activation likelihood estimation studies (43, 44), besides being close to the cathodal stimulation site of left TPJ (31, 32).

Note that, at baseline, tDCS responder and tDCS non-responder groups did not differ on AVH severity. However, tDCS responders had significantly more severe positive and negative symptoms than tDCS non-responders. Heterogeneity within the dataset [due to different tDCS techniques (conventional and HD-tDCS) and MRI data acquisition from 2 scanners] may be potential limitations (refer to Supplementary Table 1).

We have pooled neuroimaging and clinical data from two different modalities of the tDCS technique: conventional tDCS and HD-tDCS. We chose to do this because both of these methods: (a) work on the same principle, i.e., application of polarity-dependent weak intensity direct current to shift resting membrane potential, (b) had comparable stimulation protocol: cathodal stimulation of left TPJ, 2 mA current strength, 10-session spread over 5 days, and (c) targeted the same dysfunctional circuits/connectivity patterns in the brain, i.e., those implicated in auditory verbal hallucination. Though HD-tDCS is believed to overcome the pattern of diffused the electric field observed in conventional tDCS; it should be noted this belief is based on mathematical modeling and is yet to be substantiated by neuroimaging, neurophysiological and behavioral studies. Furthermore, it is still unclear from the existing proof of concept studies whether this difference in electric field distribution between these two techniques yields any discernible difference in the clinical and behavioral outcomes. Though treatment response prediction to HD-tDCS and conventional tDCS certainly deserves a nuanced, discernible approach, however, doing so was beyond the scope of the present study. The findings of this study demonstrate that baseline connectivity patterns of brain areas crucial to the experience of hallucination can predict response to therapeutic direct current stimulation techniques—both conventional tDCS and HD-tDCS, albeit the methodological differences in the respective stimulation protocol. Future studies should compare neuroimaging, neurophysiological, behavioral, and clinical measures across these two neurostimulation protocols to establish whether AVH treatment response significantly differs between these two.

Regarding the other potential limitation due to data from two different scanners, we believe that such methodological differences in data are anticipated with the advent of data pooling across research communities and multi-site studies in progress. Indeed, the success of our predictive models, despite the heterogeneity in brain stimulation technique and scanner type, indicates a promising and generalizable prognostic approach across various tDCS methodologies. Another limitation is that the study design didn’t have a placebo arm and hence doesn’t delineate add-on tDCS effects from the potential placebo effect. Future studies can utilize machine learning approaches to take a more nuanced approach toward the classification of treatment responses by further profiling placebo response from add-on tDCS treatment response.

Recent reports comparing treatment response variability in brain stimulation techniques (TMS and tDCS) have suggested a lack of variability in treatment response between sham and true groups to be a deterrent for precision medicine for brain stimulation methods (87). As the report was trans-diagnostic, and not substantiated by neurobiological evidence, we request caution in drawing conclusions. Whether “statistically insignificance” is always a proxy for “clinical significance” in the context of treatment response can be debated. For example, differences in the antipsychotic treatment response profile between a non-responder and a partial responder to antipsychotic treatment may not be statistically significant. However, this distinction is clinically meaningful enough to enlist about 20–30% of SZ patients as “treatment-resistant AVH.” In our extensive experience with tDCS (spanning a decade), we have noted distinct neurobiological differences in tDCS treatment response profiles (82) which we are continuing to investigate. The present study is one such meaningful attempt that underscores neurobiological features can predict tDCS treatment response. We acknowledge we are nowhere near a bench to bedside approach, that would enlist a substantial sample size, and intuitive algorithm(s) employing readily accessible features like demographics, clinical history, etc. alongside neurobiological features extracted from affordable investigation modalities like EEG and fNIRS. This proof-of-concept study aimed at demonstrating the feasibility of rs-fMRI measures in predicting tDCS treatment response encourages such research.

As noted earlier (88), effective optimization of stimulation protocols for non-invasive brain stimulation techniques requires accurately identifying which patients will respond to the treatment. One such endeavor is a recent study proposing electric-field modeling as a suitable method for characterizing clinical response to tDCS (89). Our attempt likewise seeks to elucidate observed variability in clinical response to tDCS. The focus of this study was to examine the effect of add-on tDCS on AVH. Hence, we chose those schizophrenia patients with persistent AVH without remission despite treatment with at least one antipsychotic medication at an adequate dose for a minimum period of 3 months along lines of selection criteria of an earlier study (90). Treatment-resistant schizophrenia is defined as non-response to treatment trials with at least two different antipsychotics of adequate dose and duration. Thus, our choice of one adequate trial with antipsychotic rather than two made more patients to qualify for this study; hence our study findings are not generalizable to patients with treatment-resistant schizophrenia.

Lastly, we are aware that studies similar to ours have worked with sample sizes far larger than ours (≥ 45 subjects) (13, 14, 16). Given that small sample sizes often produce poor results—especially when training neural network models with lots of parameters—we used “transfer learning”: here we first trained a CNN model on another large dataset (*N* = 441); we then “transferred” this learned model to our domain, by then training that model on data from our target domain. Though sample size estimation for ML approaches can be tricky and is highly influenced by the model complexity, dataset and the prediction task in general, the standard accepted approach is to train on pilot data and empirically examine the model performance. Our empirical results demonstrate that our models have successfully found patterns in the imaging features that can effectively predict treatment response (outcome label). Of course, it is always useful to explore how well this learned model will generalize, by exploring its performance on other datasets, on different cohorts.

To the best of our knowledge, this is the first functional neuroimaging study to use ML to produce a model that can identify which SZ-AVH patients will or will not respond to tDCS therapy. Wide variation in methodological parameters of tDCS-fMRI integration studies—including the time of scan relative to tDCS (pre, post, concurrent), tDCS parameters (intensity, duration, number of sessions, montage, etc.), study design and control condition, and fMRI method (BOLD, ASL, resting)—warrant replication and application of computational models to explain sources of variability (91). Perhaps future replication in a larger sample will pave the way for neurobiologically informed and pathophysiologically relevant profiling of tDCS responders and non-responders based on targeted symptoms, which in turn has the potential to advance individualized tDCS therapy and contribute to precision medicine involving brain stimulation techniques.

## Data availability statement

The data analyzed in this study is subject to the following licenses/restrictions: The datasets (including clinical data) generated during and/or analyzed during the current study are available from corresponding authors on a reasonable request. Requests to access these datasets should be directed to GV, Department of Psychiatry, National Institute of Mental Health and Neurosciences, Bangalore—560029, India, Corresponding author.

## Ethics statement

The studies involving human participants were reviewed and approved by the Institute Ethics Committee of National Institute of Mental Health And Neurosciences (NIMHANS), Bengaluru, India. The RCT tDCS study was duly registered with Clinical Trial Registry India (CTRI/2014/12/005307). The Research Ethics Board at the University of Alberta approved the secondary analysis of archived, de-identified data. The patients/participants provided their written informed consent to participate in this study.

## Author contributions

AP: methodology, software, validation, formal analysis, writing original draft, and visualization. AB: formal analysis, investigation, data curation, writing original draft, writing—review and editing, and visualization. SK: conceptualization, methodology, software, writing original draft, supervision, and funding acquisition. VS, VSS, and RP: investigation, resources, data curation, and writing—review and editing. JN: conceptualization, methodology, resources, writing—review and editing, supervision, and project administration. SD and AG: writing—review and editing and project administration. RG: writing—review and editing, supervision, project administration, and fund acquisition. GV: conceptualization, methodology, investigation, resources, writing original draft, writing—review and editing, supervision, project administration, and funding acquisition. All authors contributed to the article and approved the submitted version.

## Footnotes

1
https://web.conn-toolbox.org/

2
http://www.cma.mgh.harvard.edu/fsl_atlas.html

3
https://scikit-learn.org/stable/modules/generated/sklearn.linear_model.LogisticRegression.html

## Abbreviations

SZ: Schizophrenia
tDCS: Transcranial direct current stimulation
AVH: auditory verbal hallucinations
rs-FC: resting-state functional connectivity
LSTG: left superior temporal gyrus
CNN: convolutional neural networks
ECT: Electroconvulsive Therapy
TMS: Transcranial Magnetic Stimulation
ML: machine learning
EEG: Electroencephalography
BOLD: blood-oxygen-level-dependent
LASSO: least absolute shrinkage and selection operator
SVM: Support Vector Machine
MDD: Major depressive disorder
HD-tDCS: High definition Transcranial direct current stimulation
DLPFC: dorsolateral prefrontal cortex
TPJ: temporoparietal junction
RCT: Randomized Controlled Trial
MRI: Magnetic resonance imaging
AC-PC: Anterior commissure and posterior commissure
CGI: clinical global impression scores.
