# Supplementary material for: Superior temporal gyrus functional connectivity predicts transcranial direct current stimulation response in Schizophrenia: A machine learning study
Source: Front Psychiatry. 2022 Aug 5;13:923938. doi: 10.3389/fpsyt.2022.923938 (PMC9388779; doi:10.3389/fpsyt.2022.923938)
Supplement: Supplementary file 1 [file Data_Sheet_1.PDF]

SUPPLEMENTARY MATERIAL

METHODOLOGY

tDCS Procedures

- **Conventional tDCS procedure:** Twenty-eight SZ patients received conventional tDCS as per previous descriptions (current strength of 2-mA, duration of 20-minutes with additional ramp-up and ramp-down time of 20-seconds each, given twice-daily separated by 3-hours, for five days) (Bose et al., 2014). The electrodes were positioned as per 10-20 international electrode placement system. Anode was placed midway between F3 and Fp1 to target the left dorsolateral prefrontal cortex (DLPFC), and cathode was positioned midway between T3 and P3 to target the left temporoparietal junction (TPJ). [Note: Out of these 28 patients, 23 patients were part of an RCT tDCS study (CTRI/2014/12/005307) with open-label extension previously published (Bose et al., 2018); 13 of these 28 patients had received verum tDCS during the RCT phase while the remaining ten patients had received verum tDCS in the open-label extension phase. The remaining five patients had received open-label tDCS. One of these patients had received right-sided tDCS (anode targeting right DLPFC and cathode targeting right TPJ) (Bose et al., 2015)].

- **HD-tDCS procedure:** Eleven SZ patients received high-definition tDCS (HD-tDCS) as per previous description (Sreeraj et al., 2018). The electrodes were positioned as per 10-10 international electrode placement system. The central electrode placed at CP5 injected a direct current of -2mA while the surrounding four return electrodes (at FC3, FT7, PO7, P1) each had current strength of +0.5 mA (4x1 electrode montage). Each session was of 20 minutes duration with additional ramp-up and ramp-down time of 30-seconds each, given twice-daily separated by 3-hours, for five days.

Note: fMRI findings need to be put into context in light of recent findings where the test-retest reliability of task-based fMRI have been put to question. This doubt can be potentially extended to rest fMRI finding too though brain networks like DMN, salience, etc. have been found to be fairly consistent across demographics and seed-based correlations are less likely but potentially subject to test-retest variability. Addressing the test-retest reliability of fMRI findings is beyond the scope of this study. However, we would take the opportunity to highlight that our methodology has chosen and retained only the most consistent features

from repeated sampling via leave-one out method (10-folds) and thus has very high internal consistency reliability.

Choice of LSTG as seed region with respect to tDCS

Conventional tDCS is known to produce diffused electric field potential so it's a reasonable to say this region is stimulated by the conventional tDCS protocol. Furthermore, because of its proximity to CP5 (10-10 montage of electrode placement), this region falls within the gambit of HD-tDCS induced electric field potential as well.

Table 1: Descriptive according to tDCS type (conventional tDCS/HD-tDCS), study type (RCT/OL) and scanner (Siemens Skyra/Philips Ingenia) type

Total	RCT-ctDCS_Verum	RCT-ctDCS_OL	OL_ctDCS	OL_HDtDCS
34	13	10	3	8
**Total**	**ctDCS**	**HDtDCS**		
34	26	8		
**Total**	**Skyra**	**Ingenia**		
34	25	9		
**Total**	**153 Scans (Skyra)**	**303 Scans (Skyra)**		
**27(Final=25)**	7 (Final=6)	20 (Final=19)	2 subjects excluded at QC	

ctDCS=conventional tDCS; HD-tDCS=High definition tDCS; RCT=Randomized Control Trial; RCT_OL=Open-label arm followed by RCT; OL=Open-label (clinical); QC=Quality Check

L1 logistic regression algorithm built from demographic and clinical features

Our baseline data suggests that negative symptoms and positive symptoms were different between responders and non-responders groups. In view of this, we tested whether these clinical variables could offer similar performance as the fMRI features. Cross-validated performance of an equivalent L1-Logistic regression model using demographic and baseline clinical factors is presented below.

Table 2: Performance of models using 5 x10 fold Cross-validation -- Mean (standard error)

	Accuracy	Precision	Sensitivity	Specificity	True positive	True negative	False positive	False negative
**L1 regularized - Logistic Regression using neuroimaging data**	**72.5 (3.8)**	**74.5 (4.1)**	**78.0 (4.9)**	**67.0 (5.4)**	**13.0 (0.6)**	**11.2 (0.3)**	**5.8 (0.3)**	**4.0 (0.6)**
**CNN**	59.41 (1.93)	59.43 (1.90)	58.82 (4.07)	60.0 (3.07)	10.0 (0.69)	10.2 (0.52)	6.8 (0.52)	7.0 (0.69)
**Pretrained CNN**	68.82 (1.05)	69.63 (1.90)	68.24 (4.27)	69.41 (3.86)	11.6 (0.72)	11.8 (0.65)	5.2 (0.65)	5.4 (0.72)
**L1 regularized - Logistic Regression using baseline demographic and clinical features**	66.2 (3.9)	70.6 (4.8)	60.0 (5.8)	69.0 (5.6)	10.6 (0.8)	11.8 (0.2)	5.2 (0.2)	6.4 (0.8)

References

Bose, A., Shivakumar, V., Agarwal, S.M., Kalmady, S.V., Shenoy, S., Sreeraj, V.S., Narayanaswamy, J.C., Venkatasubramanian, G., 2018. Efficacy of fronto-temporal transcranial direct current stimulation for refractory auditory verbal hallucinations in schizophrenia: A randomized, double-blind, sham-controlled study. Schizophrenia research 195, 475-480.

Bose, A., Shivakumar, V., Narayanaswamy, J.C., Nawani, H., Subramaniam, A., Agarwal, S.M., Chhabra, H., Kalmady, S.V., Venkatasubramanian, G., 2014. Insight facilitation with add-on tDCS in schizophrenia. Schizophrenia Research 156(1), 63-65.

Bose, A., Sowmya, S., Shenoy, S., Agarwal, S.M., Chhabra, H., Narayanaswamy, J.C., Venkatasubramanian, G., 2015. Clinical utility of attentional salience in treatment of auditory verbal hallucinations in schizophrenia using transcranial direct current stimulation (tDCS). Schizophrenia research 164(1-3), 279-280.

Sreeraj, V.S., Dinakaran, D., Parlikar, R., Chhabra, H., Selvaraj, S., Shivakumar, V., Bose, A., Narayanaswamy, J.C., Venkatasubramanian, G., 2018. High-definition transcranial direct current simulation (HD-tDCS) for persistent auditory hallucinations in schizophrenia. Asian journal of psychiatry 37, 46-50.
